# Penehyclidine Hydrochloride Preconditioning Provides Cardioprotection in a Rat Model of Myocardial Ischemia/Reperfusion Injury

**DOI:** 10.1371/journal.pone.0138051

**Published:** 2015-12-02

**Authors:** Duomao Lin, Jun Ma, Yanyan Xue, Zhaoqi Wang

**Affiliations:** Center for Anesthesiology, Beijing Anzhen Hospital, Capital Medical University, Beijing, 100029, P. R. China; Indiana University School of Medicine, UNITED STATES

## Abstract

To investigate the impacts and related mechanisms of penehyclidine hydrochloride (PHC) on ischemia/reperfusion (I/R)-induced myocardial injury. A rat model of myocardial I/R injury was established by the ligation of left anterior descending coronary artery for 30 min followed by 3 h perfusion. Before I/R, the rats were pretreated with or without PHC. Cardiac function was measured by echocardiography. The activities/levels of myocardial enzymes, oxidants and antioxidant enzymes were detected. Evans blue/TTC double staining was performed to assess infarct size. Cardiomyocyte apoptosis was evaluated by TUNEL assay. The release of inflammatory cytokines and inflammatory mediators was detected by ELISA. Western blot was performed to analyze the expression of COX-2, IκB, p-IκB and NF-κB. Meanwhile, the rats were given a single injection of H-PHC before I/R. The effects of PHC on myocardial infarct and cardiac function were investigated after 7 days post-reperfusion. We found that PHC remarkably improved cardiac function, alleviated myocardial injury by decreasing myocardial enzyme levels and attenuated oxidative stress in a dose-dependent manner. Additionally, PHC preconditioning significantly reduced infarct size and the apoptotic rate of cardiomyocytes. Administration of PHC significantly decreased serum TNF-α, IL-1β, IL-6 and PGE_2_ levels and myocardium COX-2 level. Meanwhile, the expression levels of p-IκB and NF-κB were downregulated, while IκB expression was upregulated. H-PHC also exerted long-term cardioprotection in a rat model of I/R injury by decreasing infarct size and improving cardiac function. These results suggest that PHC can efficiently protect the rats against I/R-induced myocardial injury.

## Introduction

Acute myocardial infarction (AMI) is a common disease with high morbidity and mortality [1]. More than 700,000 people reportedly die from this disease every year in China [2]. AMI may cause insufficient blood supply and increase the myocardial necrosis area [3]. AMI diagnosis in old people is difficult because of dementia and dysphasia [4]. Currently, reperfusion therapy with primary percutaneous coronary intervention (PCI) effectively reduces mortality. However, this combination therapy may cause reperfusion injury [5]. Therefore, how to relieve I/R injury in the treatment of AMI is of great importance.

Penehyclidine hydrochloride (PHC) is an anti-cholinergic drug synthesized by Academy of Military Medical Sciences in China [6], which possesses both anti-muscarinic and anti-nicotinic activities. More importantly, its application almost certainly not causes M2 receptor-associated side effects to your cardiovascular system [7–9]. Previous findings have demonstrated that PHC is a promising therapeutic agent in the treatment of asthma and chronic obstructive pulmonary disease (COPD) [7,10]. Moreover, administration of PHC remarkably alleviates I/R-induced renal dysfunction [11] and cerebral injury [12,13] by inhibiting cell apoptosis and alleviating oxidative stress. However, its roles and related mechanisms in myocardial I/R injury are elusive.

In the present study, we used a rat model of I/R injury and then the rats were pretreated with three different dose regimens of PHC to explore the dose-effect relationship and probably mechanisms in therapeutics. Next, we further assessed the possible effects of H-PHC on myocardial infarct and cardiac function at day 7 post-I/R.

## Materials and Methods

### Animals and ischemia/reperfusion (I/R) rat model

Male Wistar rats (8 weeks of age) were purchased from Charles River Laboratories (Beijing, China) and were fed a standard chow diet and water *ad libitum*. The rats were housed in a constant temperature (22°C) condition with 12 h light/12 h dark cycles. The animal procedures were performed in accordance with the Guidelines for the Care and Use of Laboratory Animals of Capital Medical University, and approved by the Ethics Committee of Capital Medical University.

We performed coronary artery ligation (CAL) to induce I/R injury in Wistar rats. Before the surgery, the rats were anesthetized with 10% chloral hydrate (300 mg/kg bodyweight) by intraperitoneal injection and ventilated with a HX-300S animal respirator (Chengdu Technology & Market co., Ltd, Chengdu, China) (tidal volume, 6–8 ml/kg; ventilator frequency, 80 breaths/min). The heart was exposed via a left thoracotomy and left anterior descending (LAD) coronary artery was ligated for 30 min followed by 3 h reperfusion. The signs, including muscle tone and tail/pedal withdrawal response, were used to monitor the adequacy of the anesthesia. At the end of the experiment, the rats were sacrificed by an administration of pentobarbital sodium (200 mg/kg) by intraperitoneal injection and phlebotomy.

The rats were randomly divided into 6 groups (n = 18 in each group): (i) Sham group, the rats underwent sham surgery; (ii) Sham+H-PHC group, the rats were injected intravenously with high dose of penehyclidine hydrochloride (H-PHC; 1 mg/kg bodyweight) 30 min before sham surgery; (iii) I/R group, the rats were subjected to a 30 min LAD coronary artery ligation followed by 3 h reperfusion; (iv) I/R+L-PHC group, the rats were injected with low dose of PHC (L-PHC; 0.1 mg/kg bodyweight) 30 min before I/R; (v) I/R+M-PHC group, the rats were injected with moderate dose of PHC (M-PHC; 0.3 mg/kg bodyweight) 30 min before I/R; (vi) I/R+H-PHC group, the rats were injected with H-PHC (1 mg/kg bodyweight) 30 min before I/R.

### Echocardiography measurement

After 3 h reperfusion, Philips IE33 ultrasound system (Philips Healthcare, Amsterdam, The Netherlands) was used to measure ejection fraction (EF), fractional shortening (FS), left ventricular end-diastolic pressure (LVEDP) and left ventricular systolic pressure (LVSP).

### Determination of CK, LDH and AST activities in serum

After 3 h reperfusion, the levels of serum creatine kinase (CK), lactate dehydrogenase (LDH) and aspartate aminotransferase (AST) in serum were measured using commercial kits (Nanjing Jiancheng Bioengineering Institute, Nanjing, China) according to the manufacturer’s instructions.

### Determination of SOD activity and MDA content in serum

After 3 h reperfusion, superoxide dismutase (SOD) activity and malondialdehyde (MDA) level in serum were determined using commercial kits (Nanjing Jiancheng Bioengineering Institute) following the manufacturer’s instructions.

### Determination of total GSH, total NO level, CAT activity and GSH-Px activity in myocardial tissues

After 3 h reperfusion, the homogenated myocardial tissues were centrifuged at 12000 rpm for 10 min and the supernatant was collected. Total reduced glutathione (GSH) level was determined using GSH assay kit (Nanjing Jiancheng Bioengineering Institute) according to the manufacturer’s instructions. Total level of nitric oxide (NO) was determined using Total NO Assay Kit (Beyotime Institute of Biotechnology). The activities of glutathione peroxidase (GSH-Px) and catalase (CAT) in myocardial tissues were measured using commercial kits (Nanjing Jiancheng Bioengineering Institute).

### Measurement of ROS level

After 3 h reperfusion, myocardial tissues were collected, minced, homogenated in mitochondria isolation reagent A (Beyotime Institute of Biotechnology) containing PMSF and centrifuged at 600 g for 5 min (4°C). The supernatants were then transferred to a new tube and centrifuged at 11000 g for 10 min. The supernatants were aspirated and the pellets were lysed with mitochondria lysis buffer (Beyotime Institute of Biotechnology). The concentrations of mitochondrial proteins were determined using a bicinchoninic acid (BCA) kit (Beyotime Institute of Biotechnology). The proteins were incubated with DCFH-DA solution (1 mM) and incubated for 30 min at 37°C and then analyzed by a microplate reader ELX-800 (BioTek, Winooski, VT, USA).

### Infarct size measurement

After 3 h reperfusion, LAD coronary artery was retied. Evans blue dye (Wokai, Shanghai, China) was injected through the jugular vein. Ischemic region and area at risk (AAR) were defined. Subsequently, the hearts were removed, frozen at -20°C for 30–60 min and sliced into slices. Then, the slices were incubated with 1% TTC solution in PBS (Solarbio, Beijing, China) at 37°C in the dark and images were photographed. Myocardial infarct size was expressed as percentage of infarct area (IA) over AAR (IA/AAR×100%).

### Determination of apoptotic rate

After 3 h reperfusion, myocardial tissues were obtained, embedded in paraffin and cut into 5-mm thick slices. After deparaffinization with xylene and rehydration through graded alcohols, the sections were permeabilized in 0.1% Triton X-100 for 8 min and then blocked with H_2_O_2_ to quench endogenous peroxidase activity. Then, the sections were incubated with DAB (Solarbio) for 1 h at 37°C and dyed with hematoxylin. TUNEL-positive cells were counted under a microscope with 400× magnification (DP73; Olympus, Tokyo, Japan). The apoptotic rate was calculated as the ratio of TUNEL-positive cells to the total number of cardiomyocytes.

### Determination of TNF-α, IL-1β, IL-6 and PGE_2_ in serum

After 3 h reperfusion, blood samples were collected and serum was obtained by centrifugation. Then, serum (TNF)-α, interleukin (IL)-1β, IL-6 and prostaglandin E_2_ (PGE_2_) levels were determinated using ELISA kits (USCN, Wuhan, China) according to the manufacturer’s instructions.

### Western blot analysis

RIPA lysis buffer containing 1% PMSF was added into myocardial tissue and the supernatant was harvested by centrifugation (4°C, 12000 rpm, 10 min). Protein concentration was determined using a BCA Protein Assay Kit (Beyotime Institute of Biotechnology, Haimen, China). Protein lysates (40 μg) were separated on SDS-PAGE and transferred to PVDF membranes (Millipore, Bedford, MA, USA). Subsequently, the membranes were blocked with 5% non-fat milk/1% BSA and then incubated overnight at 4°C with cyclooxygenase (COX)-2 antibody (1:1000), IκB antibody (1:1000), NF-κB antibody (1:1000, Wanleibio, Shenyang, China) and p-IκB antibody (1:500, BIOSS, Beijing, China). Then, the membranes were incubated for 45 min at 37°C with HRP-conjugated anti-rabbit IgG antibody (Beyotime Institute of Biotechnology). Protein bands were detected using ECL agent (Wanleibio) and the band intensities were quantified using Gel-Pro-Analyzer Software (Media Cybernetics, Bethesda, MD, USA).

### Long-term effect of PHC on myocardial infarct and cardiac function

The rats were separated into three groups: The rats in the Sham group (i) and I/R group (ii) underwent the same operation as previously described. The I/R+H-PHC group (iii) rats were given a single injection of H-PHC (1 mg/kg bodyweight) 30 min before LAD ligature, and then the rats were subjected to I/R. After 7 days, the rats were anesthetized. Cardiac function was measured by echocardiography. Infarct size was determined using Evans blue/TTC staining.

### Statistical analysis

Results were expressed as the mean±SD. Data were analyzed and images were processed using GraphPad Prism 5.0 software (GraphPad Software, Inc., San Diego, CA, USA). Statistical analyses were performed with one-way analysis of variance (ANOVA) followed by Bonferroni post hoc tests. A value of *P*<0.05 was considered statistically significant.

## Results

### Effects of PHC on cardiac function

**Fig 1:**
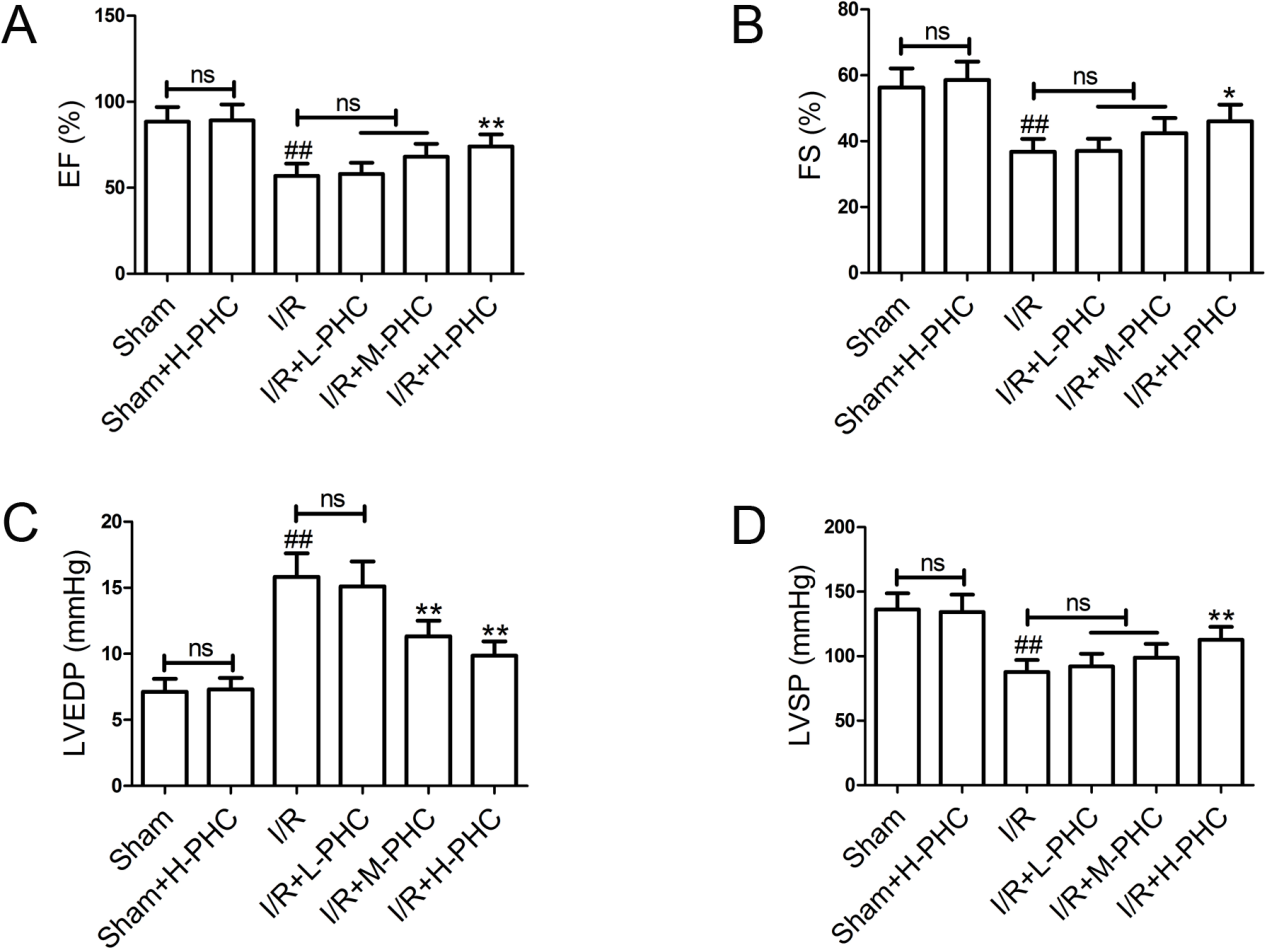
Effects of PHC on cardiac function. **(A)** EF. **(B)** FS. **(C)** LVEDP. **(D)** LVSP. Values were mean±SD (n = 6 in each group). ##*P*<0.01 vs. Sham; *P<0.05 and **P<0.01 vs. I/R (ns means no significant difference).

We investigated the impacts of PHC on cardiac function. There were no significant differences in EF (Fig 1A), FS (Fig 1B), LVEDP (Fig 1C) and LVSP (Fig 1D) between the Sham group and the Sham+H-PHC group, indicating that H-PHC administration had no adverse effects on these parameters. I/R injury greatly decreased EF, FS and LVSP, and increased LVEDP in the I/R group (*P*<0.01). However, pretreatment with H-PHC significantly elevated EF [from (56.9±7.2)% to (74.1±7.0)%, *P*<0.01], FS [from (36.8±3.9)% to (46.0±5.1)%, *P*<0.05] and LVSP [from (87.8±9.2) mmHg to (112.8±10.0) mmHg, *P*<0.01] when compared with the I/R group. Both M-PHC and H-PHC downregulated LVEDP [M-PHC: I/R group, (15.82±1.79) mmHg vs. I/R+M-PHC group, (11.33±1.17) mmHg, *P*<0.01; H-PHC: I/R group, (15.82±1.79) mmHg vs. I/R+H-PHC group, (9.85±1.09) mmHg, *P*<0.01].

### Effects of PHC on myocardial enzymes and oxidative stress markers

**Fig 2:**
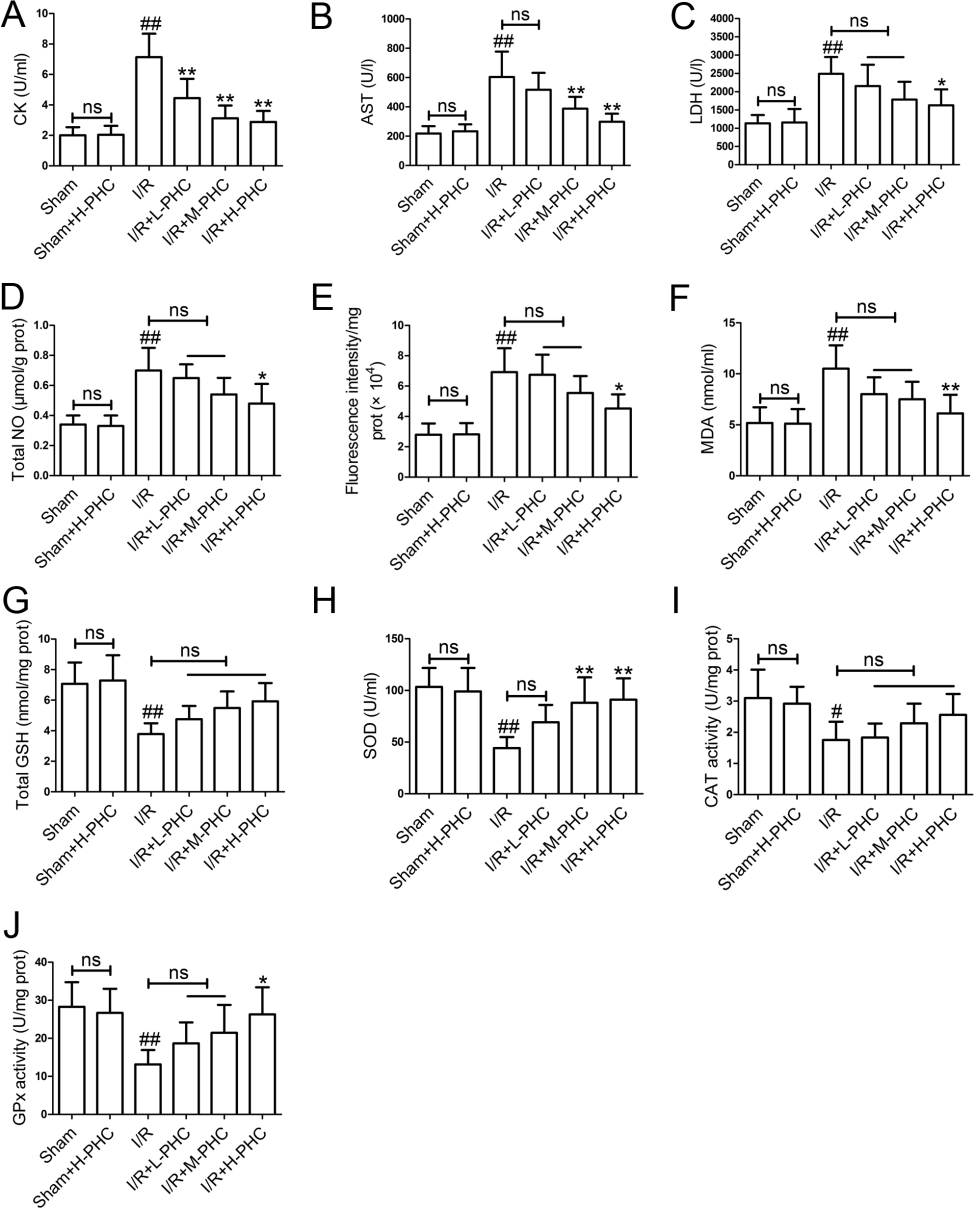
Effects of PHC on myocardial injury and oxidative stress. The rats were subjected to 30 min ischemia and 3 h reperfusion. The levels of CK **(A)**, AST **(B)**, LDH **(C)**, total NO **(D)**, ROS **(E)**, MDA **(F)** and total GSH **(G)** were measured after 3h reperfusion. The activities of SOD **(H)**, CAT **(I)** and GSH-Px **(J)** were determined. Values were mean±SD (n = 6 in each group). #*P*<0.05 and ##*P*<0.01 vs. Sham; *P<0.05 and **P<0.01 vs. I/R (ns means no significant difference).

To determine the effects of PHC on myocardial enzymes, we detected the activities of CK, AST and LDH in serum. Higher activities of CK (Fig 2A), AST (Fig 2B) and LDH (Fig 2C) were detected in the I/R group in comparison with the Sham group (*P*<0.01). However, pretreatment with PHC showed a significant reduction in the activities of all serum myocardial enzymes in a dose-dependent manner when compared with the I/R group [CK: all *P*<0.01; AST: I/R+M-PHC group and I/R+H-PHC group, *P*<0.01; LDH: I/R+H-PHC group, *P*<0.05].

To determine the effects of PHC on oxidative stress, we measured several oxidative stress markers in serum and myocardial tissues. I/R injury significantly increased total NO level (Fig 2D), ROS production (Fig 2E) and MDA content (Fig 2F) (*P*<0.01). Compared with the Sham group rats, total GSH level (a non-enzymatic antioxidant) (Fig 2G, *P*<0.01), SOD (Fig 2H, *P*<0.01), CAT (Fig 2I, *P*<0.05) and GSH-Px activities (Fig 2J, *P*<0.01) were markedly decreased in the I/R group rats. PHC pretreatment restored the all these markers in a dose-dependent manner in comparison with the I/R group [total NO: I/R+H-PHC group, *P*<0.05; ROS: I/R+H-PHC group, *P*<0.05; MDA: I/R+H-PHC group, *P*<0.01; SOD: I/R+M-PHC group and I/R+H-PHC group, *P*<0.01; GSH-Px: I/R+H-PHC group, *P*<0.05]. However, there were no significant differences in the activity of CAT and total GSH level between the treatment groups and the I/R group.

### Effects of PHC on myocardial infarct

**Fig 3:**
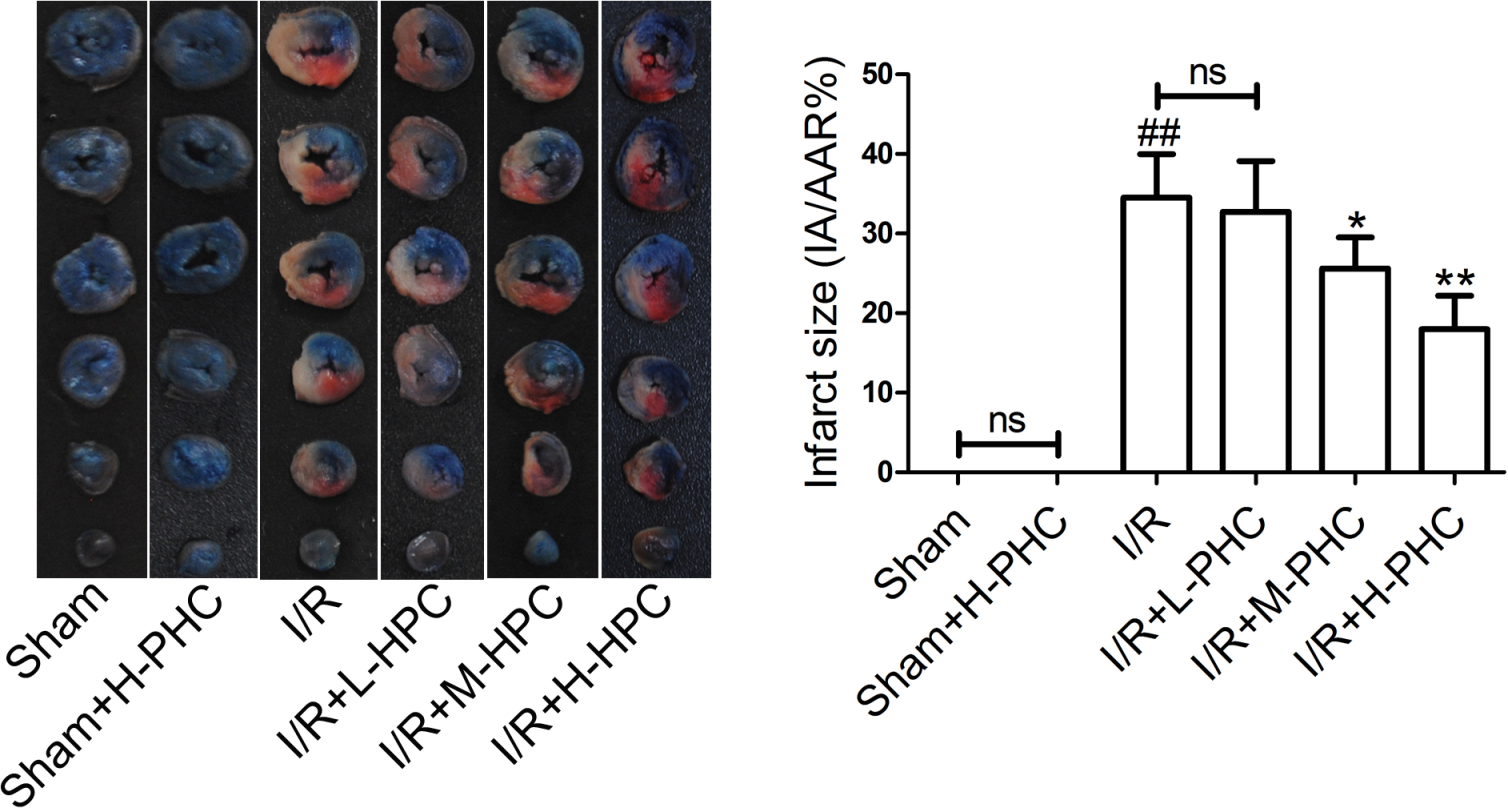
Histological analysis of myocardial infarction area. Infarct size was measured after 30 min ischemia and 3 h reperfusion. Scale bars represent 10 mm. Representative images were shown. Data are expressed as mean±SD (n = 6 in each group). ##*P*<0.01 vs. Sham; *P<0.05 and **P<0.01 vs. I/R.

As shown in Fig 3, I/R injury remarkably induced an increase in infarct size in the I/R group [(34.51±5.45)%, *P*<0.01] when compared with the Sham group. Myocardial infarct sizes were significantly reduced in M-PHC- [(25.60±3.90)%, *P*<0.05] and H-PHC-treated I/R rats [(17.98±4.21)%, *P*<0.01] compared with the I/R group rats. However, L-PHC pretreatment did not influence infarct size [I/R+L-PHC group, (32.71±6.39)% vs. I/R group, (34.51±5.45)%].

### Effects of PHC on cardiomyocyte apoptosis

**Fig 4:**
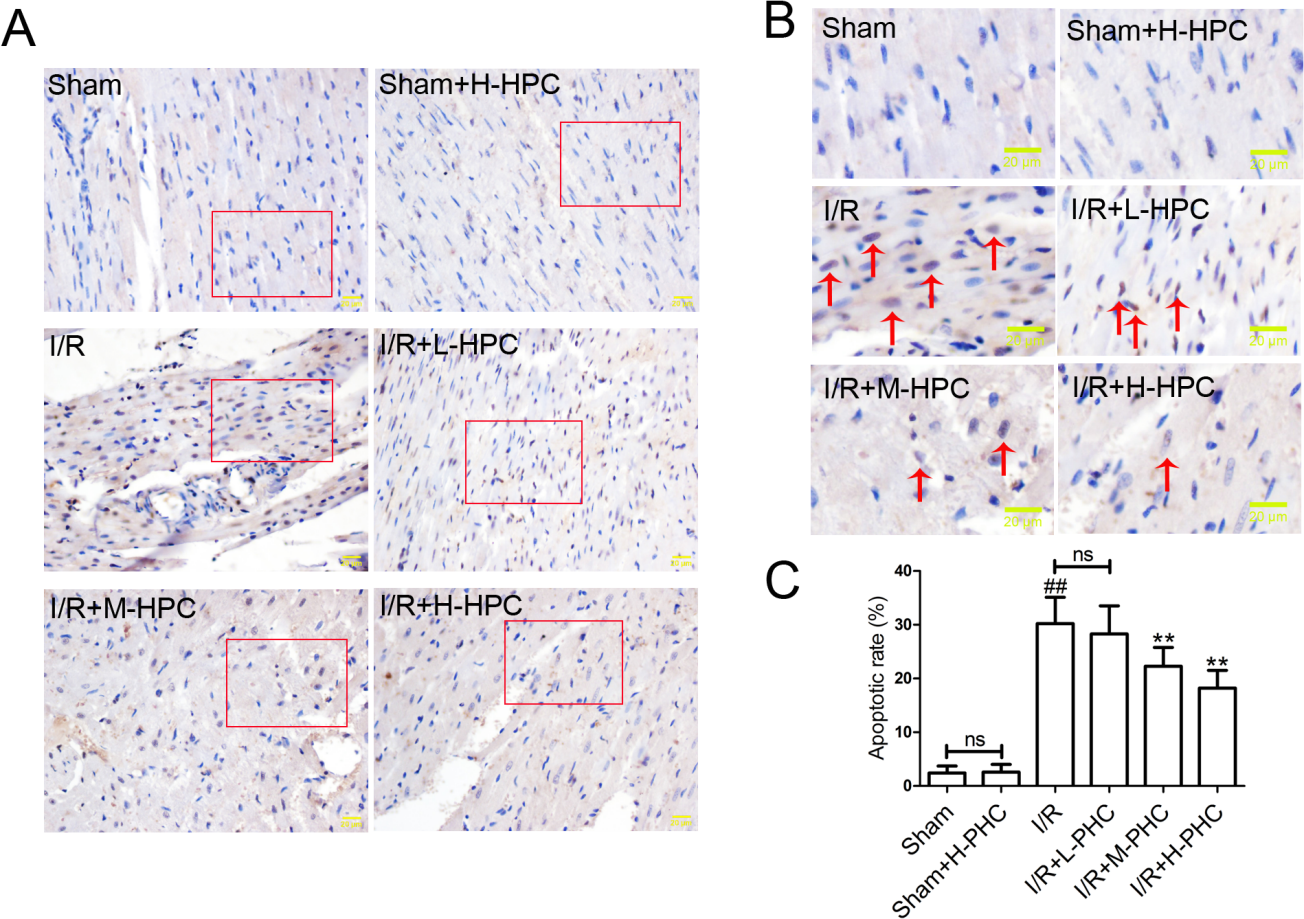
Effects of PHC on cardiomyocyte apoptosis. After reperfusion, myocardial tissues were subjected to TUNEL staining. Representative images **(A)** were photographed and showed. Images in **(B)** are insets from red squares in **(A)**. The red arrows pointed to the cells that presented typical apoptosis features. The apoptotic rates in each group were calculated **(C)**. Scale bars represent 20 μm. Values are mean±SD (n = 6 in each group). ##*P*<0.01 vs. Sham; *P<0.05 and **P<0.01 vs. I/R.

To analyze the effects of PHC on cardiomyocyte apoptosis in a rat model of I/R, we performed a TUNEL assay. The apoptotic rate was significantly increased in the I/R group compared with the Sham group [Fig 4A, 4B and 4C; Sham group, (2.4±1.3)% vs. I/R group, (30.2±4.9)%, *P*<0.01]. The apoptotic rates in the I/R+M-PHC group [(22.3±3.5)%, *P*<0.01] and I/R+H-PHC group [(18.2±3.3)%, *P*<0.01] were significantly reduced compared with the I/R group.

### Effects of PHC on inflammatory cytokines and inflammatory mediators

**Fig 5:**
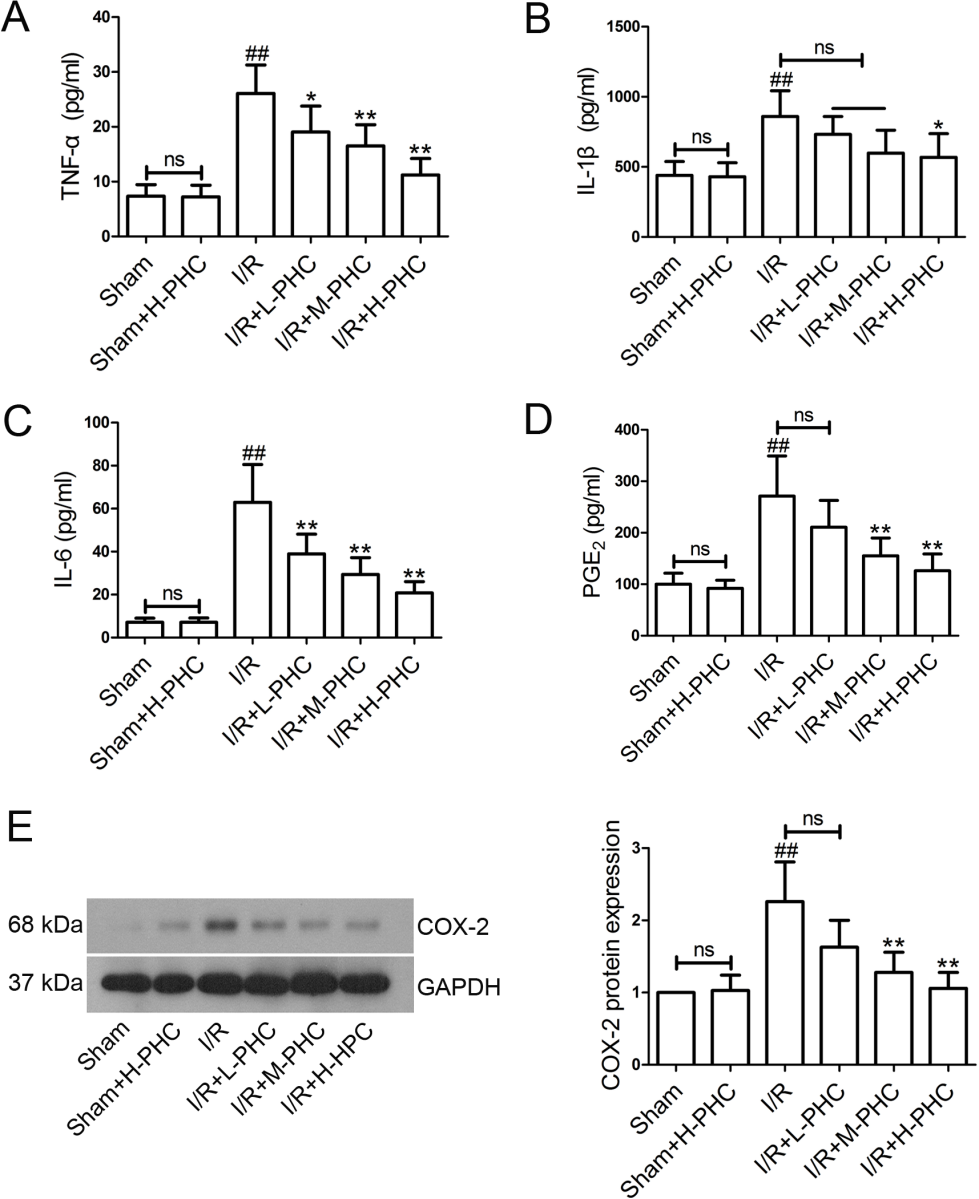
Effects of PHC on the levels of inflammatory cytokines and mediators. The levels of serum TNF-α **(A)**, IL-1β **(B)**, IL-6 **(C)** and PGE_2_ **(D)** were dectected using ELISA kits (n = 6 in each group), and myocardium COX-2 level **(E)** was measured by western blot (n = 5 in each group). Data were expressed as means±SD and analyzed by one-way ANOVA followed by Bonferroni post hoc tests. ##*P*<0.01 vs. Sham; *P<0.05 and **P<0.01 vs. I/R (ns means no significant difference).

The levels of serum TNF-α (Fig 5A), IL-1β (Fig 5B), IL-6 (Fig 5C), PGE_2_ (Fig 5D) and myocardium COX-2 (Fig 5E) in the I/R group were significantly increased in comparison with the Sham group (*P*<0.01). However, PHC pretreatment significantly reduced the TNF-α, IL-1β, IL-6, PGE_2_ and myocardium COX-2 levels in a dose-dependent manner [TNF-α: I/R+L-PHC group, *P*<0.05, I/R+M-PHC group and I/R+H-PHC group, *P*<0.01; IL-1β: I/R+H-PHC group, *P*<0.05; IL-6: all *P*<0.01; PGE_2_: I/R+M-PHC group and I/R+H-PHC group, *P*<0.01; COX-2: I/R+M-PHC group and I/R+H-PHC group, *P*<0.01].

### Effects of PHC on p-IκB, IκB and NF-κB expression

**Fig 6:**
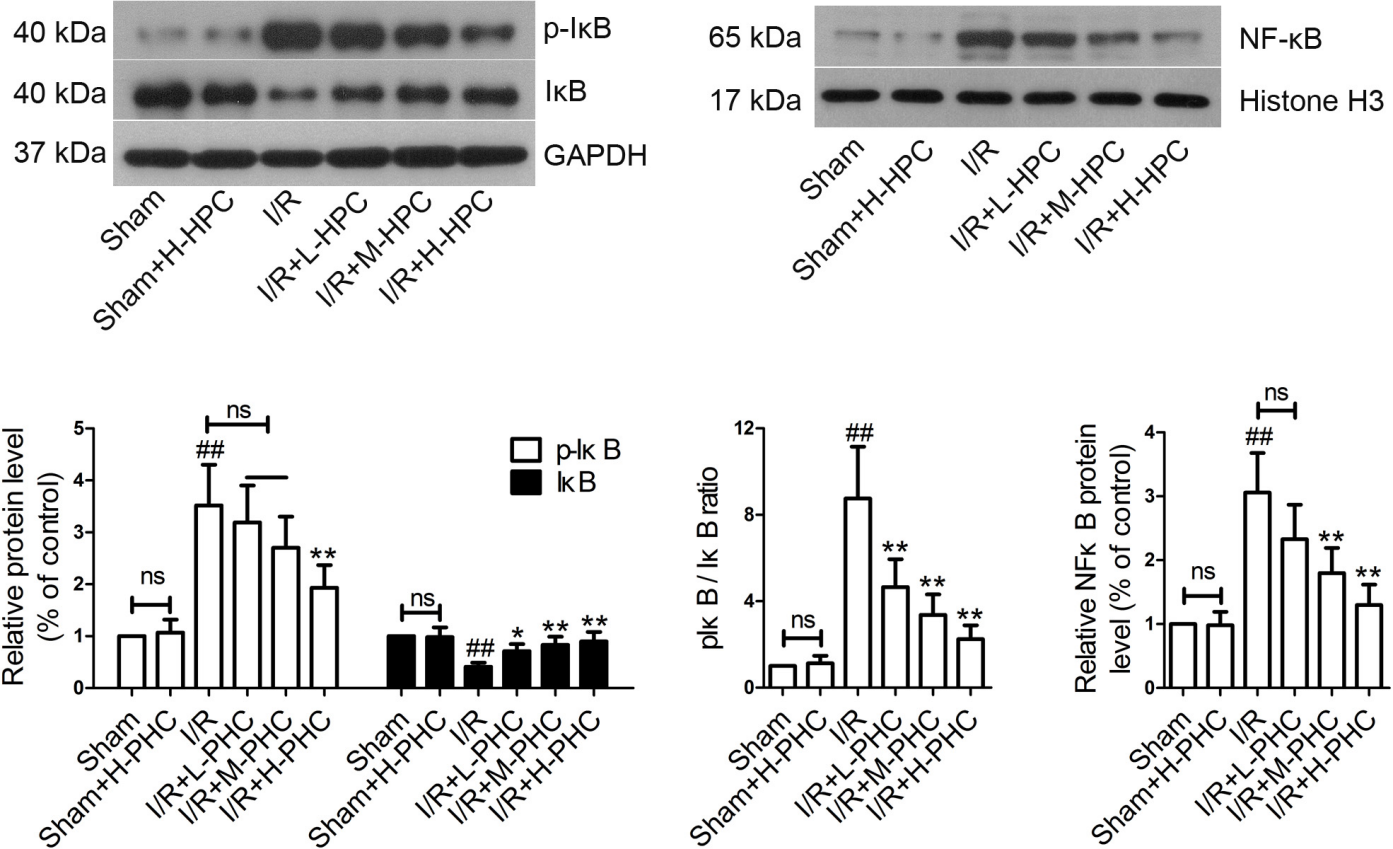
Effects of PHC on the expression of p-IκB, IκB and NF-κB in heart tissues after I/R. Myocardial tissues were obtained and the expression of p-IκB, IκB and NF-κB were analyzed by western blot. The band densities were normalized to GAPDH or Histone H3. Data were expressed as means±SD (n = 5 in each group). ##*P*<0.01 vs. Sham; *P<0.05 and **P<0.01 vs. I/R (ns means no significant difference).

We analyzed the impacts of PHC on p-IκB, IκB and NF-κB protein expression by western blot. The results showed that the levels of p-IκB and NF-κB and p-IκB/IκB ratio in the I/R group were higher and IκB expression level was lower than the Sham group (Fig 6) (*P*<0.01). However, pretreatment with PHC diminished phosphorylation of IκB and significantly decreased NF-κB expression and p-IκB/IκB ratio, while IκB expression was markedly increased [p-IκB: I/R+H-PHC group, *P*<0.01; IκB: I/R+L-PHC group, *P*<0.05, I/R+M-PHC group and I/R+H-PHC group, *P*<0.01; p-IκB/IκB ratio: all *P*<0.01; NF-κB: I/R+M-PHC group and I/R+H-PHC group, *P*<0.01].

### Long-term effects of PHC on myocardial infarct and cardiac function

**Fig 7:**
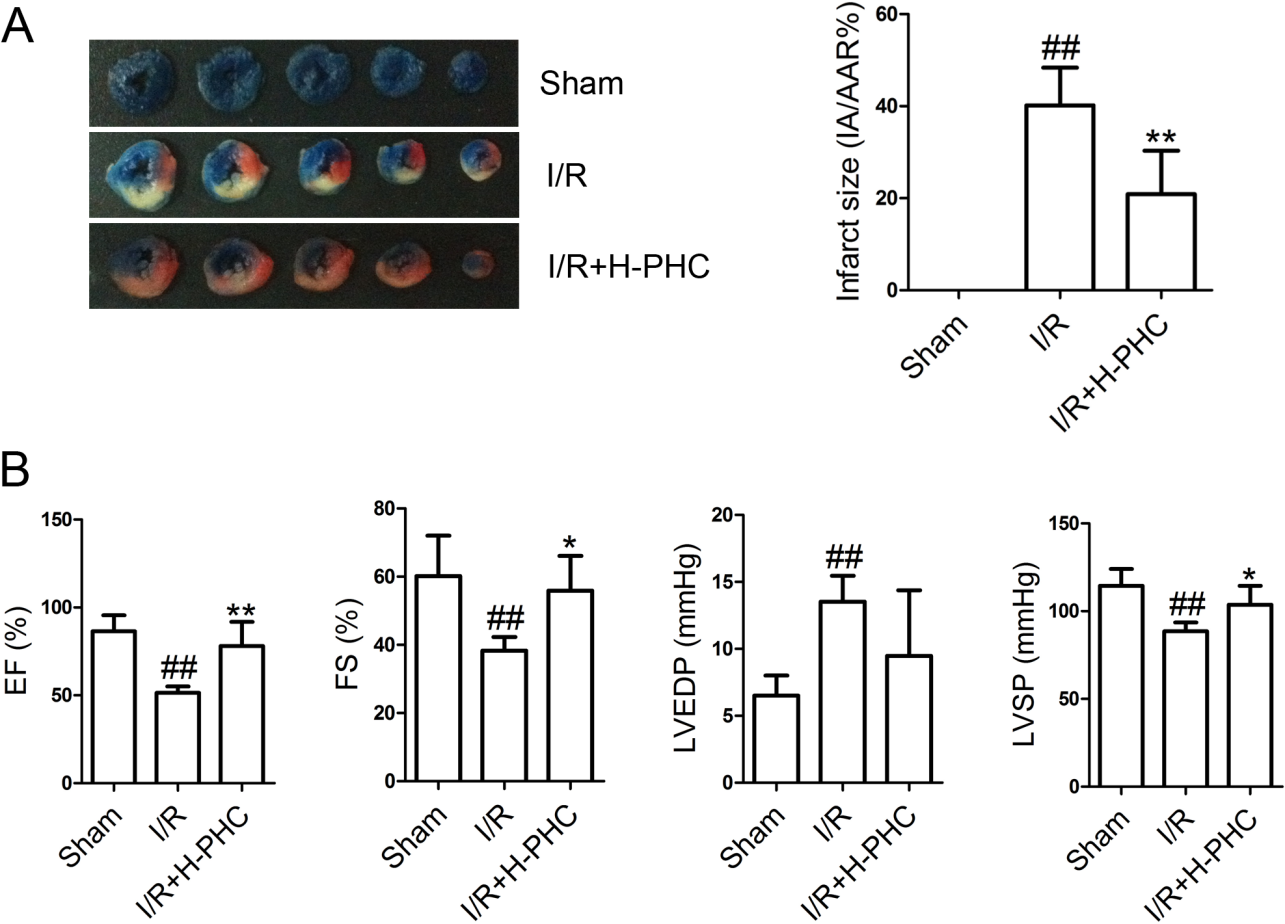
Long-term effect of H-PHC on myocardial infarct and cardiac function. The rats were given a single injection of H-PHC 30 min before the surgery, and then the rats were subjected to ischemia/reperfusion. After 7 days, infarct size **(A)** and hemodynamic changes **(B)** were measured. Scale bars represent 10 mm. Data were expressed as means±SD (n = 6 in each group). ##*P*<0.01 vs. Sham; *P<0.05 and **P<0.01 vs. I/R.

As expected, I/R injury greatly increased myocardial infarct size (Fig 7A, *P*<0.01). However, infarct size was significantly reduced at 7 d after H-PHC pretreatment (*P*<0.01). We then evaluated the long-term effects of PHC on hemodynamic parameters (Fig 7B). Compared with the Sham group, a significant decrease of EF, FS and LVSP, and an increase of LVEDP was observed in the I/R group (*P*<0.01). However, H-PHC pretreatment resulted in a significant elevation of EF (*P*<0.01), FS (*P*<0.05) and LVSP (*P*<0.05), and a downregulation of LVEDP.

## Discussion

Myocardial ischemia followed by reperfusion is always applied to the therapies of acute coronary syndrome, open heart surgery and heart transplantation. Although reperfusion is essential for the survival of ischemic tissue, reperfusion itself may cause additional injury, which is called I/R injury [14]. Penehyclidine hydrochloride (PHC), an anticholinergic agent, has great selectivity for the M1 and M3 receptor subtypes [15,16]. It has protective effect on cerebral [12] and intestinal [17] I/R injury, and other organ damage [7,18]. However, the roles of PHC in myocardial I/R injury remains unclear. In this study, we found that pretreatment with PHC improved cardiac function, suppressed myocardial injury and oxidative stress, reduced infarct size, inhibited cardiomyocyte apoptosis and inflammatory cytokine release, decreased the levels of p-IκB, NF-κB and p-IκB/IκB ratio, and increased IκB level. Moreover, H-PHC (a single injection before I/R) exerted long-term cardioprotection *in vivo*.

Cardiac function depends on the ventricular systolic and diastolic functions [19]. Recent studies have demonstrated that myocardial I/R injury may induce the changes of cardiac hemodynamic parameters and then affect cardiac function [20]. In our experiments, consistent with previous studies, the levels of EF, FS and LVSP were significantly decreased while LVEDP level was increased in I/R rats, indicating that I/R impaired cardiac function. However, EF, FS and LVSP were significantly evelated, and LVEDP level was downregulated after PHC pretreatment (especially high dose of PHC), suggesting that PHC could improve cardiac function and protect the myocardium from I/R injury.

The levels of serum myocardial enzymes (CK, AST and LDH) are used to assess the degree of myocardial injury [21]. Their elevation may associate with the occurrence of the ischemic heart disease and acute coronary syndromes [22]. In the present study, the increases of LDH, CK, AST in serum confirmed the damage to cardiocytes during I/R. Administration of PHC in three different doses caused significant changes in CK, AST and LDH activities. Oxidative stress is well known to be a major cause of tissue injury that induced by I/R. During this process, oxidants and oxygen radicals are increased, including nitric oxide (NO) and hydroxyl free radicals (HO^−^) [23]. It also has been reported that I/R injury causes the generation of ROS, which leads to tissue damage and cell death [24]. CAT, SOD and GSH-Px are antioxidant enzymes against ROS and other oxygen free radicals [25,26]. GSH is an important member of antioxidant defense system and the expression of GSH indicates the balance of oxidants and antioxidants [27]. MDA is one of the products of lipid peroxidation and is used as a marker for oxidative damage [28]. Our results demonstrated that PHC markedly reduced total NO level, ROS production and MDA content. Additionally, enhanced activities of SOD, CAT and GSH-Px, and elevated total GSH level were detected in all the PHC-treated groups, which revealed that PHC protected cardiocytes from I/R injury by reducing myocardial enzyme levels and attenuating oxidative stress [11].

Myocardial I/R-induced injury involves necrosis and apoptosis [29]. Accumulated evidences have shown that inhibiting cardiomyocyte apoptosis via therapeutic interventions can alleviate I/R injury [30–32]. We then studied the impacts of PHC on cardiomyocyte apoptosis using TUNEL assay. In this study, we found that I/R increased cardiomyocyte apoptosis, which was consistent with previous studies [33]. The results showed that PHC markedly decreased the apoptotic rate, indicating that PHC protected against I/R injury via its antiapoptotic activity.

The inflammatory responses play a major role in the pathogenesis of acute myocardial infarction (AMI) [34,35]. In addition, recent studies have demonstrated that TNF-α triggers a cytokine cascade including increasing the expression of chemokines and adhesion molecules, and then aggravates myocardial I/R injury after AMI [34,36,37]. IL-6 [38] and IL-1β [39] are also the markers of inflammatory response induced by myocardial I/R. COX-2 is an enzyme that synthesized eicosanoids and is highly expressed upon the stimulation of cytokines and LPS [40]. PGE_2_ is an inflammatory mediator regulated by COX-2 [41]. In present study, PHC significantly reduced the release of inflammatory mediators such as TNF-α, IL-1β, IL-6, PGE_2_ and COX-2 *in vitro* [42], suggesting that PHC alleviated I/R injury via its anti-inflammatory activity.

NF-κB is an important transcriptional regulatory factor and plays an important role in myocardial I/R injury [43]. I/R injury can lead to the translocation of NF-κB into the nucleus and the transcription of downstream proinflammatory cytokines and chemokines [44]. IκBα is the inhibitory protein of NF-κB. Phosphorylation and degradation of IκBα indicate the activation of NF-κB [45]. In the present study, NF-κB and p-IκB expression increased after I/R and the increases were reduced following treatment with PHC, which was consistent with previous findings [7]. These results suggest that PHC ameliorate I/R injury possibly by inhibiting the phosphorylation of IκB and nuclear translocation of NF-κB.

In the present study, we further evaluated the long-term effect of H-PHC (at 7 d after single-injection of H-PHC) on myocardial infarct and cardiac function. We found that a single injection of H-PHC significantly decreased infarct size and improved cardiac function.

Our studies demonstrated that PHC protected the myocardium from I/R injury by attenuating oxidative stress, inflammatory response and apoptosis through the inhibition of NF-κB and the activation of IκB. Moreover, H-PHC also exerted long-term cardioprotection in a rat model of I/R injury. However, the definite mechanisms underlying the effects of PHC require further study. The above results suggest that PHC can be used as a novel strategy for the therapy of myocardial I/R injury.
